# *In vitro* and *in vivo* anti-*Pseudomonas aeruginosa* activity of a scorpion peptide derivative

**DOI:** 10.3389/fmicb.2025.1622282

**Published:** 2025-07-01

**Authors:** Zhongjie Li, Jiao Zhang, Yabo Liu, Qi Dai, Shasha Li, Bo Deng, Pengfei Wu, Wanwu Li, Yanfang Dong, Pengyang Xin, Wenlu Zhang

**Affiliations:** ^1^Microbial Pathogen and Anti-Infection Research Group, School of Basic Medicine and Forensic Medicine, Henan University of Science and Technology, Luoyang, China; ^2^State Key Laboratory of Antiviral Drugs, Pingyuan Laboratory, National Medical Products Administration (NMPA) Key Laboratory for Research and Evaluation of Innovative Drug, School of Chemistry and Chemical Engineering, Henan Normal University, Xinxiang, China

**Keywords:** *Pseudomonas aeruginosa*, skin infection, food contamination, scorpion, antimicrobial peptide

## Abstract

**Introduction:**

*Pseudomonas aeruginosa* is an important opportunistic and foodborne disease-related bacterium, and the increasing antibiotic resistance of the pathogen leads to the urgent exploration of new and effective antibacterial agents. In this study, a scorpion peptide derivative HTP2 was designed.

**Methods:**

The *in vitro* anti-*P. aeruginosa* activity was evaluated using a broth microdilution assay. A mouse model of *P. aeruginosa* skin subcutaneous infection was used to evaluate the *in vivo* anti-*P. aeruginosa* activity of HTP2. The antibacterial mechanism and influence on pathogenic factors of *P. aeruginosa* of HTP2 were also investigated.

**Results:**

HTP2 could effectively inhibit the growth of *P. aeruginosa* cells with low hemolytic activity. HTP2 killed *P. aeruginosa* in a concentration-dependent manner, and could damage the membrane, induce ROS accumulation, and interact with nucleic acids. HTP2 could also inhibit biofilm formation, motility, pyocyanin production, and elastase activity of *P. aeruginosa*. In the mouse subcutaneous infection model, HTP2 significantly reduced the bacterial load of *P. aeruginosa* cells and inhibited inflammatory infiltration in the infection area.

**Conclusion:**

HTP2 could effectively kill *P. aeruginosa in vitro* and *in vivo*, and had the potential as an anti-*P. aeruginosa* agent.

## 1 Introduction

*Pseudomonas aeruginosa* is a Gram-negative bacterium that is widespread in nature, which can be found in water, soil, plant, animal, and human. It is not only a vital food-related microorganism, but also an opportunistic pathogen. As a vital food-related microorganism, *P. aeruginosa* causes a variety of contamination and spoilage of foods (such as dairy, meat, aquatic products, fresh vegetables, etc.), drinking water, and fruit juices, which can lead to intoxications or infections associated with foodborne diseases (Wu et al., 2016; Lee et al., 2020; Nahar et al., 2021; Schalli et al., 2023). As an opportunistic pathogen, *P. aeruginosa* mainly colonizes the skin and intestines of humans, and can cause skin infections, pneumonia, urinary tract infections, and sometimes result in serious systemic infections, especially in immunosuppressed individuals (Quiñones-Vico et al., 2024). Nowadays, *P. aeruginosa* can also be isolated from hospital environment, clinical instruments, medical products, and cosmetics. Thus, effective removal and killing of *P. aeruginosa* is necessary to prevent foodborne infections and opportunistic infections caused by the pathogen. However, *P. aeruginosa* can form biofilm on various surfaces, which can protect the pathogen from antibiotics and biocides (Hauser et al., 2011; Maurice et al., 2018). Moreover, antibiotic resistance also increases the difficulty of *P. aeruginosa* clearance (Kunz Coyne et al., 2022; Liao et al., 2022). Nowadays, concerns about synthetic preservatives and antibiotic-resistant food pathogens have increased significantly. Thus, developing new and effective antimicrobial agents with no side effects and food pollution potential is urgently necessary.

Antimicrobial peptides (AMPs) are usually composed of 10 to 50 amino acids, which can be identified in bacteria, fungi, plants, vertebrates, and invertebrates (Verma et al., 2024). They usually have a positive net charge and a significant proportion of hydrophobic residues (Bin Hafeez et al., 2021). Nowadays, lots of AMPs have been artificially designed based on their characteristics (Lourenço et al., 2023). According to the Collection of Anti-Microbial Peptides (CAMP), 24,243 AMPs have been recorded: 11827 natural AMPs and 12,416 synthetic AMPs. They show activities against a broad spectrum of microorganisms, including antibiotic-resistant strains, via targeting multiple sites, especially damaging the cell membrane, leading to rapid cell death (Gagat et al., 2024). Due to the specific action modes different from traditional antibiotics, AMPs exhibit a low propensity for inducing bacterial resistance (Fjell et al., 2011; Nguyen et al., 2011). Moreover, they also have the potential to inhibit biofilm formation (Shahrour et al., 2019). Currently, they have shown potential as drugs to inhibit infectious disease, and also receive special attention in food safety.

In this study, we designed several peptide derivatives based on the peptide (FWSFLAKIATKALPALFGSRKKSSSR, renamed as Helepsin in this study) from the scorpion *Hemiscorpius lepturus* (Kazemi-Lomedasht et al., 2017), among which a peptide derivative HTP2 with improved antibacterial activity against *P. aeruginosa* and decreased hemolysis was obtained. A mouse model of *P. aeruginosa* skin subcutaneous infection was used to evaluate the potential application of HTP2 as an antibacterial agent. The antibacterial mechanism and influence on pathogenic factors of *P. aeruginosa* of HTP2 were also investigated.

## 2 Materials and methods

### 2.1 Peptides and bacterial strains

The peptides used in the study were synthesized by GL Biochem (Shanghai) Ltd. with an amidated C-terminus, and the purity of the peptides was more than 95%. *P. aeruginosa* PAO1, *P. aeruginosa* ATCC27853, *P. aeruginosa* ATCC9027, and *P. aeruginosa* CCTCC93066 used in study were cultured using Luria-Bertani (LB) broth medium at 37°C.

### 2.2 Antimicrobial activity

The minimum inhibitory concentration (MIC) of the peptides against *P. aeruginosa* was measured using a broth microdilution assay recommended by the Clinical and Laboratory Standards Institute guidelines (CLSI, 2019). Briefly, exponential-phase *P. aeruginosa* cells were diluted in LB medium to 10^5^–10^6^ cfu/mL, and the peptides were dissolved and serially diluted in 0.9% saline. Then, the bacterial dilution (80 μL) and peptide dilution (20 μL) were added into sterile 96-well cell culture plates. The final concentration of each peptide was 3.13 μg/mL, 6.25 μg/mL, 12.5 μg/mL, 25 μg/mL, 50 μg/mL, and 100 μg/mL, respectively. Each concentration was conducted in triplicate. Thereafter, the plates were incubated with continuous shaking at 200 rpm for 18–24 h at 37°C. The lowest peptide concentration with no bacterial growth was determined as the MIC.

### 2.3 Hemolytic activity

Hemolytic activity was used to evaluate the *in vitro* toxicity of the peptides, using the method described previously (Yuan et al., 2022). Briefly, fresh mouse red blood cells (mRBCs) were washed with and then resuspended in 0.9% saline to 2% (v/v), and the peptides were dissolved and serially diluted in 0.9% saline. Then, the mRBCs suspension (100 μL) and peptide dilution (100 μL) were added into sterile 96-well cell culture plates. The final concentration of each peptide was 25 μg/mL, 50 μg/mL, and 100 μg/mL, respectively. Each concentration was conducted in triplicate, and 1% Triton X-100 and no peptide (0.9% saline) treatment were set as the positive and negative controls, respectively. Thereafter, the plates were incubated with continuous shaking at 200 rpm for 1 h at 37°C. After incubation, the plates were centrifuged at 1,000 g for 10 min, and 100 μL of the supernatant was transferred to a new 96-well plate, and the absorbance was measured at 490 nm. Hemolytic activity was evaluated using the following equation: Hemolysis% = (H_sample_ − H_negative_)/(H_positive_ − H_negative_) × 100%. H: absorbance at 490 nm.

### 2.4 Stability assay

The stability of the peptide was determined using the method described previously (Mirzaei et al., 2022; Yuan et al., 2022). For the thermal stability assay, the peptide was incubated at 60°C for 24 h. Then, the MIC of the peptide against *P. aeruginosa* PAO1 was measured. For salt stability assay, the exponential phase *P. aeruginosa* PAO1 cells were washed with and diluted in NaCl-free LB medium to 10^5^–10^6^ cfu/mL with specific amounts of NaCl, CaCl_2_, MgCl_2_, or KCl, respectively. The peptide was dissolved and serially diluted in sterile water. Then, the bacterial dilution (80 μL) and peptide dilution (20 μL) were added into sterile 96-well cell culture plates. The final concentration of each peptide was 3.13 μg/mL, 6.25 μg/mL, 12.5 μg/mL, 25 μg/mL, 50 μg/mL, and 100 μg/mL, respectively. Each concentration was conducted in triplicate. The final concentration of NaCl, CaCl_2_, MgCl_2_, and KCl was 150 mM/300 mM, 1 mM/2 mM, 1 mM/2 mM, and 2 mM/4 mM, respectively. Thereafter, the MIC of the peptide against *P. aeruginosa* PAO1 was measured.

### 2.5 Time-killing kinetics

The time-killing kinetics assay of the peptide against *P. aeruginosa* PAO1 was performed using the method described previously (Li et al., 2020). Briefly, exponential-phase *P. aeruginosa* PAO1 cells were diluted in LB medium to 10^5^–10^6^ cfu/mL, and the peptide was dissolved and serially diluted in 0.9% saline. Then, the bacterial dilution (400 μL) was incubated with peptide dilution (100 μL) at 37°C with continuous shaking at 200 rpm. The final concentration of the peptide was 12.5 μg/mL, 25 μg/mL, and 50 μg/mL, respectively. No peptide (0.9% saline) treatment served as a negative control. Aliquots were collected at 0 min, 15 min, and 30 min, serially diluted in 0.9% saline, and plated on LB agar plates. The plates were incubated at 37°C for 18–24 h, and the CFU was counted.

### 2.6 Confocal laser-scanning microscopy

The action site of the peptide on *P. aeruginosa* was determined using the method described previously (Li et al., 2014). Briefly, exponential-phase *P. aeruginosa* PAO1 cells were diluted in LB medium to 10^6^–10^7^ cfu/mL, and the FITC-labeled peptide was dissolved in 0.9% saline. Then, the bacterial dilution was incubated with the FITC-labeled peptide dilution at a volume ratio of 4:1. The final concentration of the FITC-labeled peptide was 10 μg/mL. After incubation for 20 min at 37°C, the cells were washed with and resuspended in PBS, and then immobilized on a glass slide. The cells were observed using a confocal laser-scanning microscope.

### 2.7 LPS competition binding assay

The interaction of the peptide with LPS was evaluated using the method described previously (Cao et al., 2012). Briefly, exponential-phase *P. aeruginosa* PAO1 cells were diluted in LB medium to 10^5^–10^6^ cfu/mL, and the peptides were dissolved and serially diluted in 0.9% saline containing LPS. Then, the bacterial dilution (80 μL) and peptide dilution (20 μL) were added into sterile 96-well cell culture plates. The final concentration of each peptide was 3.13 μg/mL, 6.25 μg/mL, 12.5 μg/mL, 25 μg/mL, 50 μg/mL, and 100 μg/mL, respectively. Each concentration was conducted in triplicate. The final concentration of LPS was 100 μg/mL, 200 μg/mL, or 500 μg/mL, respectively. Thereafter, the plates were incubated with continuous shaking at 200 rpm for 18–24 h at 37°C. The MIC of the peptide in the presence of LPS was determined.

### 2.8 Membrane permeability assay

The influence of the peptide on the membrane integrity of *P. aeruginosa* cells was evaluated using the method described previously (Li et al., 2016). Briefly, exponential-phase *P. aeruginosa* PAO1 cells were washed with and diluted in 0.9% saline to 10^6^–10^7^ cfu/mL, and then incubated with SYTOX green (final concentration: 5 μM) in the dark for 10 min at 37°C. The peptide was dissolved and serially diluted in 0.9% saline. Then, the bacterial dilution (50 μL) and peptide dilution (50 μL) were added into a Costar 96-well flat-bottom black plate. The final concentration of the peptide was 6.25 μg/mL, 12.5 μg/mL, and 25 μg/mL, respectively. Each concentration was conducted in triplicate, and no peptide (0.9% saline) treatment was served as a negative control. Thereafter, the fluorescence was measured every 2 min at the excitation and emission wavelengths of 488 and 525 nm, respectively.

### 2.9 Membrane potential assay

The influence of the peptide on the membrane potential of *P. aeruginosa* cells was evaluated using the method described previously (Lee and Lee, 2014). Briefly, exponential-phase *P. aeruginosa* PAO1 cells were washed with and diluted in 0.9% saline to 10^6^–10^7^ cfu/mL, and then incubated with DiBAC4(3) (final concentration: 10 μM) in the dark for 10 min at 37°C. The peptide was dissolved and serially diluted in 0.9% saline. Then, the bacterial dilution (50 μL) and peptide dilution (50 μL) were added into a Costar 96-well flat-bottom black plate. The final concentration of the peptide was 6.25 μg/mL, 12.5 μg/mL, and 25 μg/mL, respectively. Each concentration was conducted in triplicate, and no peptide (0.9% saline) treatment was served as a negative control. Thereafter, the fluorescence was measured every 2 min at the excitation and emission wavelengths of 488 and 525 nm, respectively.

### 2.10 ROS measurements

The influence of the peptide on ROS generation of *P. aeruginosa* cells was evaluated using the method described previously (Shi et al., 2021). Briefly, exponential-phase *P. aeruginosa* PAO1 cells were washed with and diluted in 0.9% saline to 10^6^–10^7^ cfu/mL, and then incubated with DCFH-DA (final concentration: 10 μM) in the dark for 20 min at 37°C. The peptide was dissolved and serially diluted in 0.9% saline. Then, the bacterial dilution (50 μL) and peptide dilution (50 μL) were added into a Costar 96-well flat-bottom black plate. The final concentration of the peptide was 6.25 μg/mL, 12.5 μg/mL, and 25 μg/mL, respectively. Each concentration was conducted in triplicate, and no peptide (0.9% saline) treatment was served as a negative control. Thereafter, the fluorescence was measured every 10 min at the excitation and emission wavelengths of 488 and 525 nm, respectively.

### 2.11 Nucleic acid binding assay

The interaction of the peptide with nucleic acids was evaluated using the method described previously (Li Z. et al., 2022). Briefly, approximately 300 ng of DNA (plasmid pET-28a) or RNA (*P. aeruginosa* PAO1 RNA) was incubated with varying concentrations of the peptide for 10 min at room temperature. Then, the mixtures were electrophoresed in a 1% agarose gel. The migration of nucleic acids in the gel was visualized using a Bio-Rad Gel Documentation system.

### 2.12 Effects on biofilm formation

The influence of the peptide on biofilm formation of *P. aeruginosa* was evaluated using the method described previously (Artini et al., 2022). Briefly, exponential-phase *P. aeruginosa* PAO1 cells were diluted in LB medium to 10^5^–10^6^ cfu/mL, and 200 μL of the bacterial dilution was added into sterile 96-well cell culture plates. After incubation for 4 h at 37°C, the wells were washed with PBS to remove the non-adherent cells. Then, 200 μL of LB medium containing varying concentrations of the peptide was added to the wells. The final concentration of the peptide was 6.25 μg/mL, 12.5 μg/mL, and 25 μg/mL, respectively. Each concentration was conducted in triplicate, and no peptide (0.9% saline) treatment was served as a negative control. After static cultivation for another 24 h at 37°C, biomasses were quantified using a crystal violet staining assay.

### 2.13 Motility assay

The influence of the peptide on the motility of *P. aeruginosa* was evaluated using the method described previously (She et al., 2018; Chen et al., 2020). Briefly, exponential-phase *P. aeruginosa* PAO1 cells were diluted in LB medium to 10^6^–10^7^ cfu/mL. To investigate swimming motility, 0.3% LB agar medium (1 mL) supplemented with varying concentrations of the peptide was poured into sterile 24-well cell culture plates. Then, 2 μL of the bacterial dilution was spotted in the center of the agar plate. After incubation for 18–24 h at 37°C, the swimming zone was observed and pictured with a camera. To investigate swarming motility, 0.5% LB agar medium (1 mL) supplemented with varying concentrations of the peptide was poured into sterile 24-well cell culture plates. Then, 2 μL of the bacterial dilution was spotted in the center of the agar plate. After incubation for 18–24 h at 37°C, the swarming zone was observed and pictured with a camera.

### 2.14 Pyocyanin assay

The influence of the peptide on the pyocyanin production of *P. aeruginosa* was evaluated using the method described previously (Pan et al., 2023). Briefly, exponential-phase *P. aeruginosa* PAO1 cells were diluted in LB medium to 10^6^–10^7^ cfu/mL, and the peptides were dissolved and serially diluted in 0.9% saline. Then, the bacterial dilution was incubated with the peptide dilution at a volume ratio of 4:1. The final concentration of each peptide was 6.25 μg/mL and 12.5 μg/mL, respectively. Each concentration was conducted in triplicate, and no peptide (0.9% saline) treatment was served as a negative control. After incubation for 16 h at 37°C with continuous shaking at 200 rpm, pyocyanin was extracted using 0.6 mL of chloroform from 1 mL supernatant of the bacterial culture. Then, the organic layer was extracted using 0.2 mL of HCl (0.2 N), and the absorbance of the upper layer was measured at 520 nm.

### 2.15 Elastase assay

The influence of the peptide on the elastase of *P. aeruginosa* was evaluated using the method described previously (Musthafa et al., 2012; Xu et al., 2015). Briefly, exponential-phase *P. aeruginosa* PAO1 cells were diluted in LB medium to 10^6^–10^7^ cfu/mL, and the peptides were dissolved and serially diluted in 0.9% saline. Then, the bacterial dilution was incubated with the peptide dilution at a volume ratio of 4:1. The final concentration of each peptide was 6.25 μg/mL and 12.5 μg/mL, respectively. Each concentration was conducted in triplicate, and no peptide (0.9% saline) treatment was served as a negative control. After incubation for 6 h at 37°C with continuous shaking at 200 rpm, the cultures were centrifuged at 12,000 r/min for 10 min at 4°C. Then, the supernatant was filtered using a 0.22 μm syringe filter. Thereafter, the filtered supernatant (100 μL) was incubated with ECR solution (900 μL: ECR 20 mg/mL, 0.1 M Tris–HCl, 1 mM CaCl_2_, pH 7.2) for 4 h at 37°C with continuous shaking at 200 rpm. After incubation, the reaction mixture was incubated on ice for 10 min, and then centrifuged at 12,000 r/min for 10 min at 4°C. The absorbance of the supernatant was measured at 520 nm.

### 2.16 Animals and subcutaneous infection model

Male BALB/c mice (20–30 g), obtained from Henan SKBEX Biology Co., Ltd., were maintained under a standard condition of humidity (50 ± 5%), temperature (25 ± 2°C), and dark–light cycles (12 h each) with free access to food and water. All the animal-associated experiments were conducted following the Animal Care and Ethics guidelines with protocols approved by the Animal Care and Use Committee of Henan University of Technology and Science. At the end of the experiments, all the mice were humanely euthanized by intraperitoneal injection of excessive pentobarbital.

A mouse subcutaneous infection model was used to evaluate the *in vivo* anti-*P. aeruginosa* activity of the peptide according to the method previously described (Li S. et al., 2022). Briefly, exponential-phase *P. aeruginosa* PAO1 cells were washed with and resuspended in 0.9% saline to approximately 10^9^ CFU/mL. Each mouse was subcutaneously injected with 50 μL of the bacterial suspension at the back near the tail. Then, the mice were randomly divided into three groups (six mice per group). One hour after infection, 50 μL of 0.9% saline (negative control), peptide solution (500 μg/mL in 0.9% saline), or ciprofloxacin solution (positive control, 500 μg/mL in 0.9% saline) was subcutaneously injected into the infected area of each mouse in the corresponding group. All the treatments were administered once a day continuously for 3 days. On the 4^th^ day, the mice were humanely euthanized, and the infected area of each mouse was sterilized using 10% povidone/iodine solution and 70% ethyl alcohol after shaving the fur. Thereafter, half of the skin abscesses were excised and homogenized in 0.9% saline. After being serially diluted in 0.9% saline, the homogeneous samples were cultured on LB agar at 37°C for 18–24 h. The number of colony-forming units (CFU) per gram of the skin abscess was subsequently calculated. Another half of the samples were used for hematoxylin and eosin staining, and the infiltration of inflammatory cells was observed.

### 2.17 Statistical analysis

The data were expressed as mean ± standard error of the mean (SEM) and analyzed using the GraphPad Prism 6 software. Differences between groups were analyzed using one-way ANOVA.

## 3 Results

### 3.1 Peptides and *in vitro* antibacterial activity

**Figure 1:**
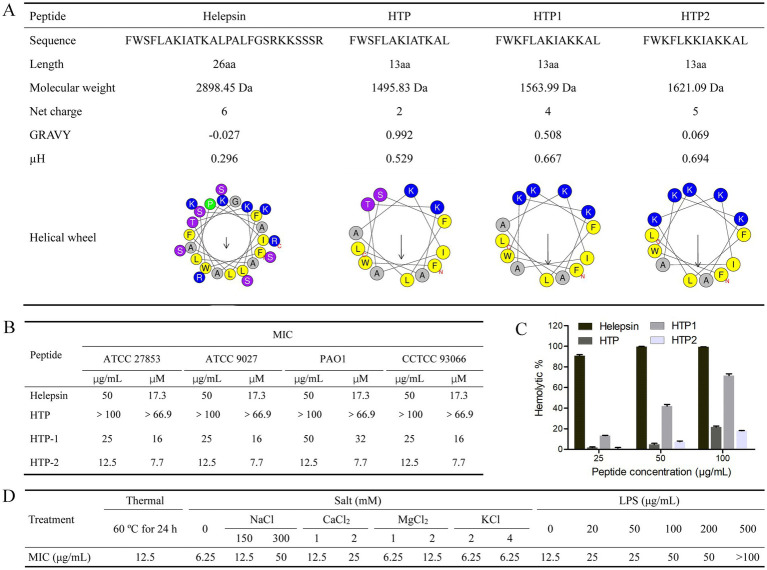
Peptides and *in vitro* activities. **(A)** Characterization and helical wheel diagram. **(B)** Anti-*P. aeruginosa* activity. **(C)** Hemolytic activity. **(D)** Stability and competition binding assay. GRAVY (Grand average of hydropathicity): Determined by ProtParam (https://web.expasy.org/protparam/). μH (Hydrophobic moment) and helical wheel diagram: Determined by the Heliquest (https://heliquest.ipmc.cnrs.fr/cgi-bin/ComputParams.py).

Based on the peptide Helepsin, three peptide derivatives (HTP, HTP1, and HTP2) were designed (Figure 1A). HTP was a truncated peptide from Helepsin; HTP1 and HTP2 were designed based on HTP by amino acid substitution with lysine. As shown in Figure 1B, the MICs of Helepsin against the tested *P. aeruginosa* strains were all 17.3 μM, while HTP had no inhibitory effects against the tested *P. aeruginosa* strains at 66.9 μM. The MICs of HTP1 against the tested *P. aeruginosa* strains were close to that of Helepsin, which were 16–32 μM. HTP2 displayed an improved activity against the tested *P. aeruginosa* strains compared to that of Helepsin with the MICs all 7.7 μM.

### 3.2 Hemolytic activity of the peptides

Hemolytic activity was used to evaluate the *in vitro* toxicity of the peptides. As shown in Figure 1C, Helepsin showed about 91% hemolysis at the concentration of 25 μg/mL (8.6 μM), while HTP showed only about 22% hemolysis at the concentration of 100 μg/mL (66.9 μM), and HTP2 showed only about 18% hemolysis at the concentration of 100 μg/mL (61.7 μM). Although HTP1 only showed about 13% hemolysis at the concentration of 25 μg/mL (16 μM), it showed about 72% hemolysis at the concentration of 100 μg/mL (63.9 μM).

### 3.3 Peptide stability of HTP2

According to the antibacterial activity and hemolytic activity, HTP2 was selected, and its stability was evaluated. As shown in Figure 1D, there are no changes in the MICs for HTP2 against *P. aeruginosa* after incubation at 60°C for 24 h. Thus, HTP2 had good thermal stability. When treated with HTP2 with different salts at varying concentrations, MICs showed varying degrees of changes. Low concentration monovalent ions (2 mM KCl, 4 mM KCl) had little effect on the antibacterial activity of HTP2, but high concentration monovalent ions (300 mM NaCl) had a greater impact on the antibacterial activity of HTP2. And low concentrations of divalent cations (1 mM/2 mM CaCl_2_, 2 mM MgCl_2_) have a significant impact on activity. Therefore, the impact of different ions, especially cations, on the activity of HTP2 varies.

### 3.4 Interaction between LPS and HTP2

Electrostatic interaction mediates the activity of AMPs, and LPS is the main anionic component on the surface of *P. aeruginosa* cells. Thus, the interaction between HTP2 and LPS was determined. As shown in Figure 1D, the MICs of HTP2 increased in the presence of additional LPS, indicating a decrease in the activity of HTP2 against *P. aeruginosa*. These results indicated that HTP2 could interact with LPS.

### 3.5 Time-killing kinetics of HTP2

**Figure 2:**
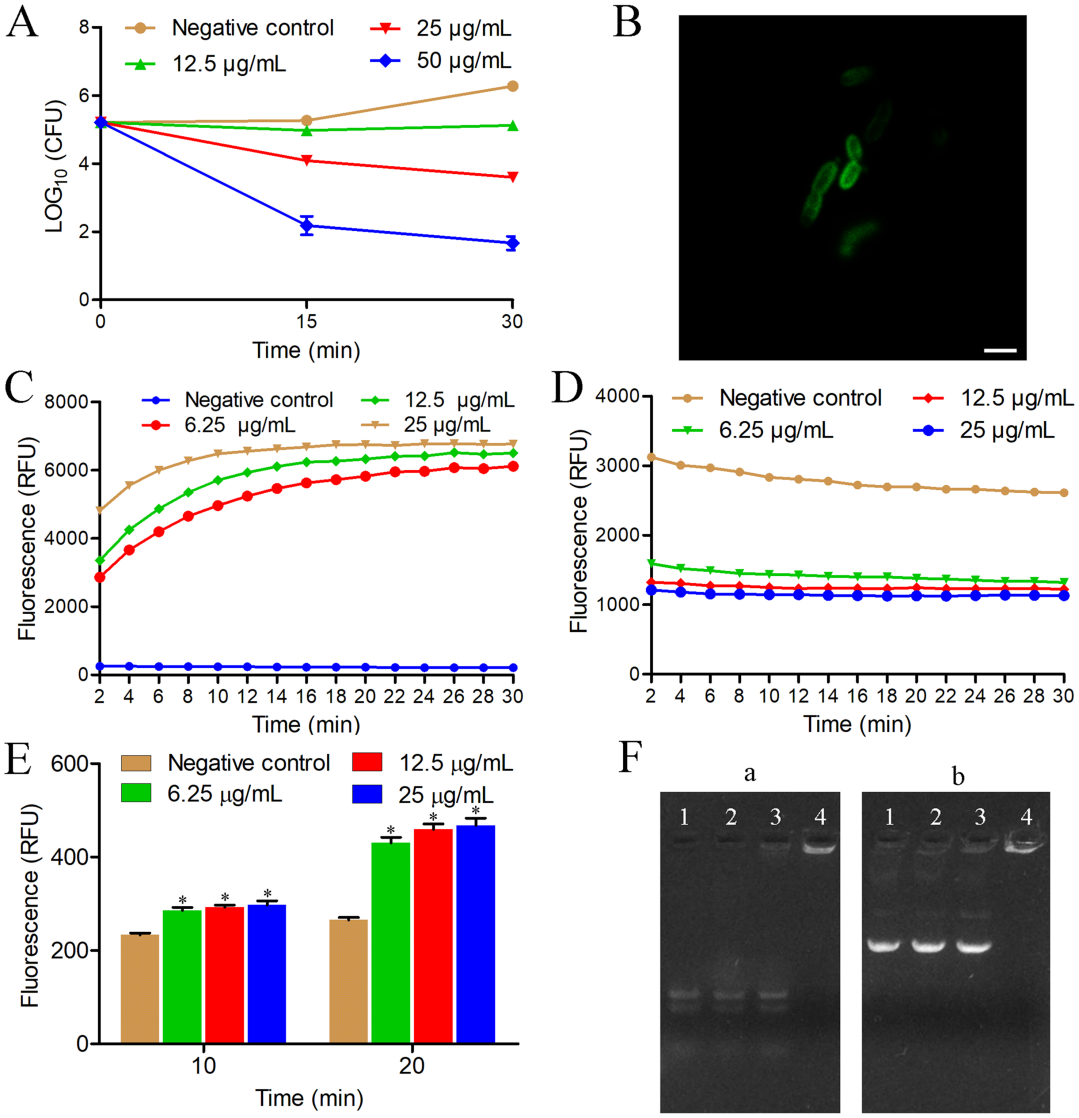
Anti-*P. aeruginosa* mechanism of HTP2. **(A)** Time-killing kinetics. Negative control group, 0.9% saline. **(B)** Confocal fluorescence microscopic images of *P. aeruginosa* cells treated with FITC-HTP2. **(C)** Membrane integrity measurement. Negative control group, 0.9% saline. RFU: Relative fluorescence unit. **(D)** Membrane potential measurement. Negative control group, 0.9% saline. RFU: Relative fluorescence unit. **(E)** ROS measurement. Negative control group, 0.9% saline. RFU: Relative fluorescence unit. *p < 0.05. **(F)** Nucleic acids binding assay. **(a)** *P. aeruginosa* RNA; **(b)** pET-28a; Ratio of peptide/nucleic acids: line 1 (0:1), line 2 (5:1), line 3 (10:1), line 4 (20:1).

To gain insights into the killing mode of HTP2 against *P. aeruginosa*, the time-killing kinetics were performed. As shown in Figure 2A, the amount of *P. aeruginosa* PAO1 cells did not show any changes after being treated with HTP2 at a concentration of 12.5 μg/mL for 30 min. When treated with HTP2 for 15 min at the concentrations of 25 μg/mL and 50 μg/mL, the amount of *P. aeruginosa* PAO1 cells decreased by about 1-log and about 3-log, respectively. Thus, HTP2 killed *P. aeruginosa* cells in a concentration-dependent manner.

### 3.6 Action sites of HTP2

To determine the action sites of HTP2, *P. aeruginosa* PAO1 cells were treated with FITC-HTP2. As shown in Figure 2B, a strong fluorescence distribution appeared on the cell surface, while there was a weak fluorescence distribution inside the cell. These results indicated that FITC-HTP2 mainly bound to the surface of *P. aeruginosa* PAO1 cells, and it could also act inside the cell.

### 3.7 Effects of HTP2 on the membrane integrity of *Pseudomonas aeruginosa*

To evaluate the influence of HTP2 on the membrane integrity of *P. aeruginosa* cells, the membrane permeability assay was performed using the fluorescence dye SYTOX green. As shown in Figure 2C, there is a significant increase in fluorescence intensity within a few minutes after treating *P. aeruginosa* PAO1 cells with HTP2 at the concentration of 6.25 μg/mL, 12.5 μg/mL, or 25 μg/mL, respectively. These results indicated that the membrane integrity of *P. aeruginosa* cells was disrupted by HTP2.

### 3.8 Effects of HTP2 on the membrane potential of *Pseudomonas aeruginosa*

To evaluate the influence of HTP2 on the membrane potential of *P. aeruginosa* cells, the membrane potential assay was performed using the fluorescence dye DiBAC4(3). As shown in Figure 2D, there is a rapid decrease in fluorescence intensity after treating *P. aeruginosa* PAO1 cells with HTP2 at the concentration of 6.25 μg/mL, 12.5 μg/mL, or 25 μg/mL, respectively. These results indicated that the membrane potential of *P. aeruginosa* cells was disrupted by HTP2.

### 3.9 Effects of HTP2 on ROS production of *Pseudomonas aeruginosa*

To evaluate the influence of HTP2 on the production of ROS in *P. aeruginosa* cells, the amount of ROS was detected using the fluorescence dye DCFH-DA. As shown in Figure 2E, there is a significant increase in fluorescence intensity after treating *P. aeruginosa* PAO1 cells with HTP2 at the concentration of 6.25 μg/mL, 12.5 μg/mL, or 25 μg/mL for 10 min or 20 min, respectively. These results indicated that HTP2 induced the production of ROS in *P. aeruginosa* cells.

### 3.10 Interaction between nucleic acid and HTP2

To evaluate whether HTP2 could interact with nucleic acids, the migration of nucleic acid in gel in the presence of HTP2 was tested. As shown in Figure 2F, the migration of RNA (Figure 2Fa) and plasmid DNA (Figure 2Fb) is retarded by HTP2. These results indicated that HTP2 could interact with different types of nucleic acids.

### 3.11 Effects of HTP2 on biofilm formation of *Pseudomonas aeruginosa*

**Figure 3:**
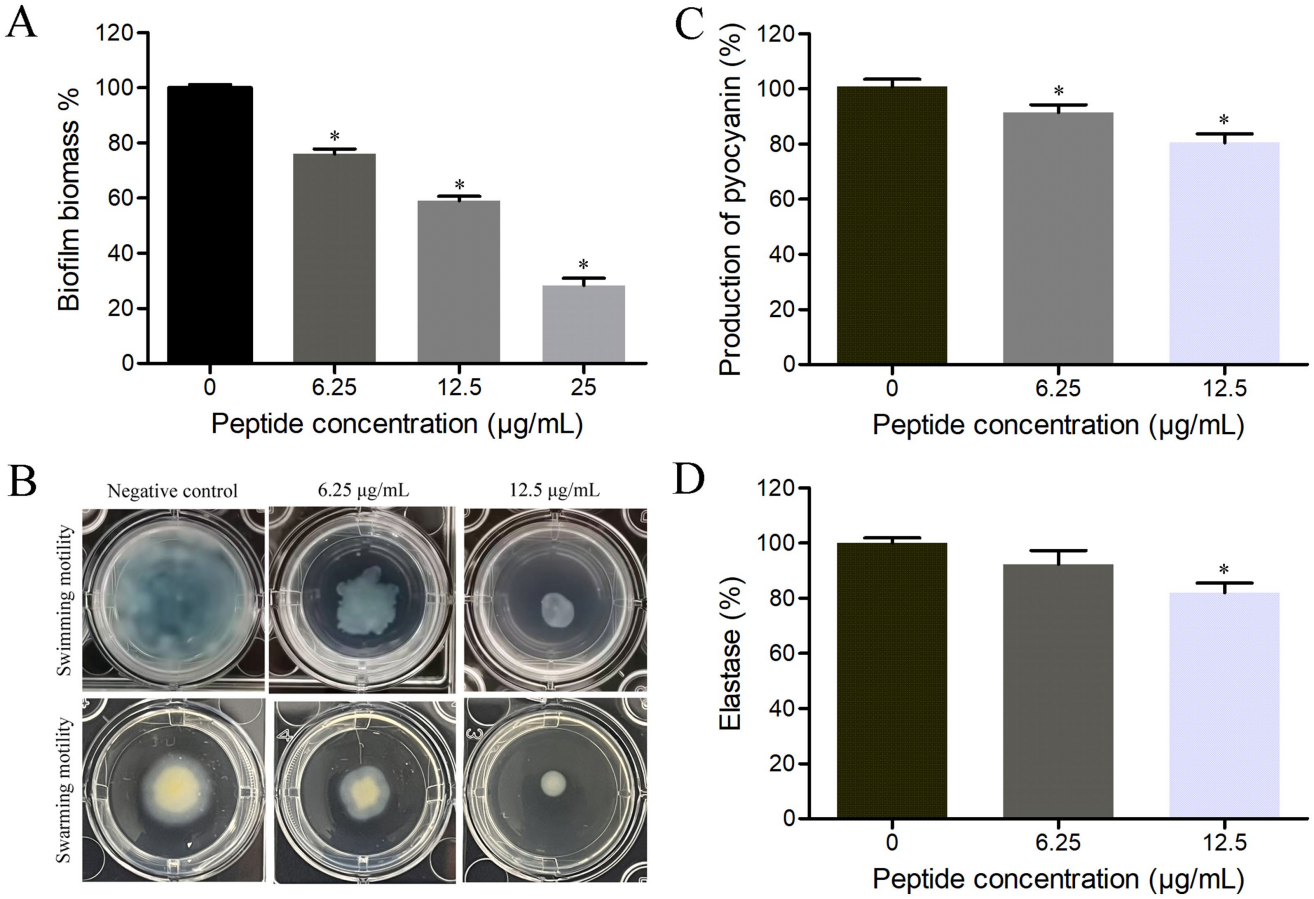
Influence of HTP2 on pathogenic factors of *P. aeruginosa*. **(A)** Biofilm formation. *p < 0.05. **(B)** Swimming motility assay. **(C)** Pyocyanin production. *p < 0.05. **(D)** Elastase activity. *p < 0.05.

Biofilm is an important pathogenic factor for *P. aeruginosa*, therefore, the influence of HTP2 on biofilm formation of *P. aeruginosa* was evaluated. As shown in Figure 3A, after treatment with HTP2 at the concentration of 6.25 μg/mL, 12.5 μg/mL, and 25 μg/mL, the biomass of *P. aeruginosa* biofilms was reduced by about 24.1% (*p* < 0.05), 41% (*p* < 0.05), and 71.7% (*p* < 0.05), respectively. Thus, HTP2 could significantly inhibit the biofilm formation of *P. aeruginosa*.

### 3.12 Effects of HTP2 on motility of *Pseudomonas aeruginosa*

Motility ability plays a crucial role in the adhesion, colonization, and invasion processes of bacteria with flagella, therefore, the influence of HTP2 on the motility ability of *P. aeruginosa* was evaluated. As shown in Figure 3B, after treatment with HTP2 at the concentrations of 6.25 μg/mL and 12.5 μg/mL, the size of the bacterial lawn area was significantly smaller than that of the negative control (non-HTP2) treatment, respectively. Thus, HTP2 could significantly inhibit the motility ability of *P. aeruginosa*.

### 3.13 Effects of HTP2 on pyocyanin production of *Pseudomonas aeruginosa*

Pyocyanin can enhance the pathogenicity of *P. aeruginosa* through multiple modes, therefore, the influence of HTP2 on pyocyanin production of *P. aeruginosa* was evaluated. As shown in Figure 3C, after treatment with HTP2 at the concentrations of 6.25 μg/mL and 12.5 μg/mL, the production of pyocyanin decreased by about 9.6% (*p* < 0.05) and 19.6% (*p* < 0.05), respectively. Thus, HTP2 could significantly inhibit the pyocyanin production of *P. aeruginosa*.

### 3.14 Effects of HTP2 on elastase of *Pseudomonas aeruginosa*

Elastase plays an important role in the invasiveness of *P. aeruginosa*, therefore, the influence of HTP2 on the elastase production/activity of *P. aeruginosa* was evaluated. As shown in Figure 3D, after treatment with HTP2 at the concentrations of 6.25 μg/mL and 12.5 μg/mL, the elastase production/activity decreased by about 7.7% (*p* > 0.05) and 18.1% (*p* < 0.05), respectively. Thus, HTP2 could inhibit the production/activity of the elastase of *P. aeruginosa*.

### 3.15 *In vivo* anti-*Pseudomonas aeruginosa* activity of HTP2

**Figure 4:**
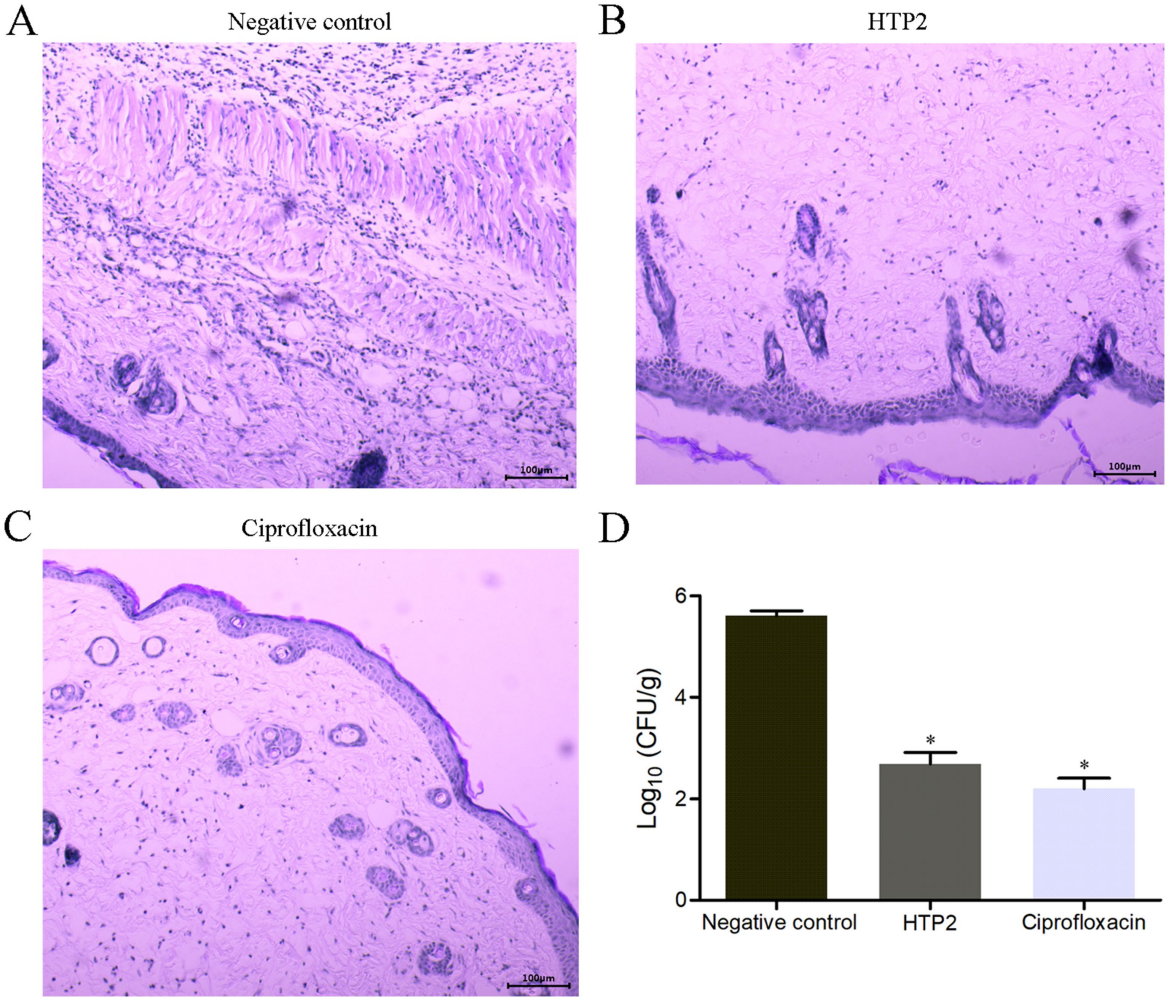
*In vivo* anti-*P. aeruginosa* activity of HTP2. **(A)** Hematoxylin–Eosin staining for the negative control group. **(B)** Hematoxylin–Eosin staining for the HTP2 treatment group. **(C)** Hematoxylin–Eosin staining for the Ciprofloxacin treatment group. **(D)** CFU per gram of the tissue. Negative control group, 0.9% saline. *p < 0.05.

A mouse subcutaneous infection model was used to evaluate the activity of HTP2 against *P. aeruginosa* under physiological conditions. As shown in Figure 4D, the bacterial load of *P. aeruginosa* from the infection area of HTP2-treated mice and Ciprofloxacin-treated mice was significantly decreased (*p* < 0.05) compared to that from the negative control group. And there was no significant difference between the HTP2-treated group and the Ciprofloxacin-treated group. Moreover, HTP2 significantly inhibited inflammatory infiltration (Figure 4B) in the infection area compared to that of the negative control group (Figure 4A). Thus, HTP2 had good anti-*P. aeruginosa* activity under physiological conditions.

## 4 Discussion

As one of the widely distributed bacteria, *P. aeruginosa* can cause food contamination, opportunistic infections, and foodborne diseases (Nahar et al., 2021; Li X. et al., 2022; Quiñones-Vico et al., 2024). The increasing antibiotic resistance of *P. aeruginosa* has made its impact more severe and its elimination more difficult (Reynolds and Kollef, 2021; Antimicrobial Resistance Collaborators, 2022). Thus, the development of novel antimicrobial agents that are safe, effective, and environmentally friendly is both critically important and urgently needed. Among these candidates, AMPs have attracted great attention.

In this study, the scorpion peptide Helepsin showed anti-*P. aeruginosa* activity with the MICs of 17.3 μM (Figure 1B), but it had a high hemolytic activity (90.8% hemolysis) at the concentration of 8.7 μM (25 μg/mL) (Figure 1C). To decrease the hemolytic activity, maintain/increase the anti-*P. aeruginosa* activity and reduce the cost, a truncated peptide HTP was designed based on Helepsin, and then two derivatives (HTP1 and HTP2) were designed based on HTP using amino acid substitution with lysine (Figure 1A). Previous studies have shown that an increase in the proportion of hydrophobic amino acids, hydrophobic moment, or net positive charge can enhance the antibacterial activity of AMPs (Brogden, 2005). Although HTP had a lower hemolytic activity compared to Helepsin (Figure 1C), it did not show anti-*P. aeruginosa* activity at the concentration of 66.9 μM (Figure 1B). It was mainly due to the decrease in percentage of hydrophobic amino acids (a higher GRAVY) and net positive charge, even with a higher hydrophobic moment (Figure 1A). Both HTP1 and HTP2 had an increased anti-*P. aeruginosa* activity, which was mainly due to the increase in proportion of hydrophobic amino acids (decrease in GRAVY), hydrophobic moment, and net positive charge (Figure 1A) compared to HTP. Moreover, HTP2 also had a very low hemolytic activity among the four peptides (Figure 1C). Thus, HTP2 was the optimal peptide in this study, which was selected for the *in vivo* study. In the mouse cutaneous infection model, HTP2 could significantly (*p* < 0.05) reduce the bacterial load of *P. aeruginosa* cells (Figure 4D) and inhibit inflammatory infiltration (Figure 4B) in the infection area. Thus, HTP2 had the potential as an antibacterial agent or a sanitizer against *P. aeruginosa*.

To investigate the mechanism of action of HTP2 against *P. aeruginosa*, *P. aeruginosa* PAO1 was selected as the model bacterial strain. Our results showed that HTP2 killed *P. aeruginosa* cells in a dose-dependent way (Figure 2A) and mainly bound to the surface of the cells (Figure 2B). It’s well known that cationic AMPs can disrupt the cytoplasmic membrane after binding to the surface of the bacterial cell, and the binding is mediated by electrostatic interactions between the peptide and anions on the cell surface (Lohner, 2009; Fjell et al., 2011). To verify whether the binding of HTP2 to *P. aeruginosa* cells was related to electrostatic attraction, the cation stability assay and LPS competition binding assay were performed. Our results showed that external cations (especially multivalent cations) and external LPS could decrease the anti-*P. aeruginosa* of HTP2 (Figure 1D), indicating that electrostatic interactions played an important role in the anti-*P. aeruginosa* activity of HTP2, and HTP2 interacted with LPS (the major anionic components on the cell surface of *P. aeruginosa*). To further verify whether HTP2 affected the integrity and function of cell membranes, the membrane permeability assay and membrane potential assay were performed. Our results showed that HTP2 could disrupt the integrity (Figure 2C) and potential (Figure 2D) of *P. aeruginosa* membrane, which would finally disrupt the normal physiological functions of the cell membranes. Besides acting on the cell membrane, AMPs can interfere with intracellular physiological regulation in various ways, such as interacting with intracellular targets and inducing ROS accumulation (Nicolas, 2009). Our results showed that HTP2 could induce the accumulation of ROS (Figure 2E), which might damage DNA, RNA, proteins, and membrane lipids (Borisov et al., 2021). HTP2 could also interact with nucleic acids (Figure 2F), which might inhibit the replication, transcription, and translation of nucleic acids. Thus, HTP2 killed *P. aeruginosa* cells via a multi-mode manner.

Many virulence factors of *P. aeruginosa* play important roles during the pathogenic processes, or are beneficial for contaminating food, or make it difficult to be eliminated. *P. aeruginosa* can form biofilm on the surface of tissue, food, or objects, which protects the pathogen from being killed by the host immune system and/or antibacterial agents (Li X. et al., 2022; Yin et al., 2022). Motility can promote bacterial adhesion and invasion of *P. aeruginosa*, help the pathogen migrate to favorable environments, and escape from harmful environments (Matilla et al., 2021; Muggeo et al., 2023). Pyocyanin, an extracellular secreted redox-active phenazine secondary metabolite produced by *P. aeruginosa*, can induce the generation of reactive oxygen species and activate pro-inflammatory signaling pathways of host cells, and can disrupt macrophage phagocytic function (Lew et al., 2025). Elastase is an extracellular secreted metalloprotease, which can promote host invasion and immune evasion of *P. aeruginosa* by cleaving the host substrates and immune system components, and can activate pathogenicity-associated proteins/proteases/exotoxins (Camberlein et al., 2022). Our results showed that HTP2 could inhibit the biofilm formation (Figure 3A), motility (Figure 3B), pyocyanin production (Figure 3C), and elastase activity (Figure 3D) of *P. aeruginosa*. Thus, HTP2 could prevent the pathogenic process of opportunistic infections and foodborne diseases caused by *P. aeruginosa* via inhibiting various pathogenic factors.

## 5 Conclusion

In conclusion, the peptide HTP2 effectively inhibited the growth of *P. aeruginosa in vitro* and *in vivo*. HTP2 killed *P. aeruginosa* cells via a multi-manner, including damaging the membrane, inducing ROS accumulation, and interacting with nucleic acids. HTP2 could also inhibit biofilm formation, motility, pyocyanin production, and elastase activity of *P. aeruginosa*. Taken together, HTP2 had the potential as an antibacterial agent or a sanitizer against *P. aeruginosa*.

## Data Availability

The raw data supporting the conclusions of this article will be made available by the authors, without undue reservation.
